# Prediction of symptomatic improvement after exposure-based treatment for irritable bowel syndrome

**DOI:** 10.1186/1471-230X-13-160

**Published:** 2013-11-19

**Authors:** Brjánn Ljótsson, Erik Andersson, Perjohan Lindfors, Jeffrey M Lackner, Karin Grönberg, Katarina Molin, Johanna Norén, Karin Romberg, Evelyn Andersson, Timo Hursti, Hugo Hesser, Erik Hedman

**Affiliations:** 1 Department of Clinical Neuroscience, Division of Psychology, Karolinska Institutet, Nobels väg 9, Stockholm 171 65, Sweden; 2 Department of Clinical Neuroscience, Division of Psychiatry, Karolinska Institutet, Stockholm, Sweden; 3 Department of Gastroenterology, Sabbatsbergs Hospital, Stockholm, Sweden; 4 Department of Medicine Huddinge, Karolinska Institutet, Stockholm, Sweden; 5 Department of Medicine, University at Buffalo School of Medicine and Biomedical Science, ECMC, Buffalo, NY, USA; 6 Department of Psychology, Uppsala University, Uppsala, Sweden; 7 Department of Behavioural Sciences and Learning, Linköping University, Linköping, Sweden; 8 Department of Clinical Neuroscience, Osher Center for Integrative Medicine, Karolinska Institutet, Stockholm, Sweden

**Keywords:** Irritable bowel syndrome, Exposure, Internet, Cognitive behavior therapy, Psychological treatment, Prediction analysis

## Abstract

**Background:**

Several studies show that psychological treatments relieve symptoms for patients suffering from irritable bowel syndrome (IBS). However, there are no consistent findings that show what patient characteristics make a psychological treatment more or less likely to result in improvement. We have previously conducted a study of a newly developed internet-delivered cognitive behavioral therapy (ICBT) that emphasized exposure to IBS symptoms and IBS-related situations and reduced symptom-related avoidance. The study showed that the treatment led to improvement in IBS symptoms compared to a waiting list and that treatment gains were maintained over a 15–18 month follow-up period. The aim of the present study was to investigate several possible predictors of short- and long-term treatment outcome in terms of symptom improvement, based on data collected in the previously conducted treatment trial.

**Methods:**

Demographics, comorbid psychological distress, IBS-related fear and avoidance behaviors, and IBS-related disability were investigated as predictors of treatment outcome in the sample consisting of 79 participants diagnosed with IBS who had undergone 10 weeks of ICBT. Predictors that were significantly correlated with symptom levels at post-treatment and follow-up were entered into multiple regression analyses that controlled for pre-treatment symptom levels.

**Results:**

There were measures within each domain, i.e., comorbid psychological distress, IBS-related fear and avoidance behaviors, and IBS-related disability, with the exception of demographic data, that were correlated with the symptom levels at post-treatment and follow-up. However, when these were entered into a multiple regression analyses that controlled for pre-treatment levels, none remained a significant predictor of the post-treatment and follow-up symptomatic status.

**Conclusions:**

The study did not find any individual characteristics that made patients more or less likely to respond to the exposure-based ICBT. The finding that comorbid psychological distress did not predict outcome is in accordance with previous studies. Reliable predictors for response to any type of psychological treatment for IBS remain to be established.

## Background

Irritable bowel syndrome (IBS) affects about 11% of the population [1], is characterized by recurring abdominal pain and diarrhea and/or constipation [2] and associated with decreased quality of life [3] and increased health-care consumption [4]. Several different types of psychological treatments have been studied as interventions for IBS, including cognitive behavior therapy (CBT), psychodynamic therapy, and hypnotherapy [5]. These treatments generally show beneficial effects but treatment effects vary considerably between studies of the same treatment types [6], and little is known about what patient characteristics predict successful outcome from psychological treatment. More knowledge in this regard is important as it can contribute to a better matching of patients to treatments and could ultimately be used to increase the total proportion of participants who respond to treatment [7].

The observation that many patients with IBS present with comorbid psychiatric conditions or psychological distress [8], which are associated with increased symptom severity [9], is often used as rationale for the application of psychological treatments for IBS [6]. Based on this rationale for psychological treatment, it would be reasonable to assume that presence of psychiatric comorbidity or high psychological distress would imply suitability, i.e., better treatment response, for psychological interventions. However, results from prediction analyses have been contradictory. We have found four studies that reported a positive association between psychological distress at pre-treatment and post-treatment improvement [10-13]. Eight studies have reported the reverse association, i.e., that psychological distress predicted worse outcome [14-21]. Finally, three studies reported no association between psychological distress and outcome [22-24]. The measures of psychological distress and the predicted outcome have differed between studies (not all have used symptomatic improvement as outcome), and the studies may therefore not be completely comparable. Notwithstanding the different methods, there does not seem to be much data backing up the common proposition that patients with IBS should benefit from psychological treatments just because they display psychological distress e.g., [5].

However, although most psychological treatments for IBS, in accordance with the common rationale, target comorbid psychological distress [6], there are also cognitive behavioral treatments that target disorder-specific behavioral patterns in IBS. During the last decade, several studies of CBT based on behavioral exposure have been conducted where the primary target of treatment has been IBS-related avoidance and worry rather than comorbid psychological distress [25-30]. This behavioral pattern, often referred to as gastrointestinal symptom-specific anxiety (GSA) [31], is associated with increased symptom severity and decreased quality of life [32,33]. The exposure-based treatments have consistently produced large and stable effect sizes on measures of IBS symptoms and quality of life [25-30]. Thus, the treatments that target GSA seem to be an exception from the rule that psychological treatments for IBS show large between-study variation in treatment effects. Based on the model employed in these treatments targeting GSA, it would be reasonable to assume that patients with IBS who show high levels of GSA, more specifically IBS-related avoidance behaviors, worry about IBS symptoms, and disability related to IBS, could be suitable candidates for treatments targeting this symptom-specific pattern. We are only aware of one study that has investigated the predictive value of GSA and disability on outcome after an exposure-based treatment. This study included a consecutively recruited sample of clinical patients with IBS and did not find that high pre-treatment levels of GSA and disability were related to larger improvement in IBS symptoms after an internet-delivered cognitive behavioral treatment (ICBT) based on exposure exercises [25]. However, these analyses were based on a small sample with a large proportion of missing data, as 7 of 30 (23%) participants in the treatment arm did not complete the post-treatment data, limiting the statistical power to detect meaningful associations and possibly introducing bias in the prediction analyses. Another previous trial of ICBT for IBS [27], had less missing data and several possible predictors were measured before treatment, including psychiatric comorbidity, GSA, and disability. Furthermore, the entire sample was assessed at a long-term follow-up [34], allowing for prediction of both the short- and long-term treatment effects. The present predictor study is based on data from that trial.

The aim of the present study was to investigate the role of psychological distress, GSA and IBS-related disability as predictors of short- and long-term symptomatic improvement following treatment with ICBT for IBS. Based on the inconsistent findings in the IBS research on outcome prediction we did not formulate any specific hypotheses.

## Methods

### Design

This study included 79 self-referred participants with a diagnosis of IBS who had participated in a randomized controlled study (ClinicalTrials.gov: NCT01171053) of ICBT for IBS, where they were randomized to either treatment (n = 39) or waiting list control (n = 40) for 10 weeks ( [27]. After the 10-week post-treatment assessment participants on the waiting list were crossed over to receive treatment and subsequently completed a post-treatment assessment after 10 additional weeks. In addition, both groups participated in a long-term follow-up assessment [34]. Since both groups were followed up simultaneously, the time between treatment completion and follow-up assessment was 18 months for the treatment group and 15 months for the group who received treatment after having been on the waiting list. In the present study, the two samples were pooled together to predict the symptomatic changes from pre-treatment to post-treatment and from pre-treatment to follow-up, based on pre-treatment characteristics. This study was approved by the Regional ethical review board in Stockholm (No: 2007/1451 - 31/4).

### Participants

All participants had received a clinical diagnosis of IBS before inclusion and fulfillment of Rome III criteria [2] was confirmed before inclusion. Inclusion criteria, recruitment procedure and participant characteristics are detailed in the original report [27]. Because 6 of 85 participants in the original trial withdrew from the study before the post-treatment assessment, this study included the remaining 79 (93%) who had participated in the post-treatment (*n* = 76) and/or the follow-up assessment (*n* = 74), and thus provided improvement data for analysis in the present study. Sample characteristics are presented in Table 1.

**Table 1:** Pre-treatment characteristics and correlations with outcome variables

				**Correlation with GSRS-IBS**	
	***n***	***m***	***sd***	**Post-treatment**	**Follow-up**
*Outcome variables*					
GSRS-IBS at post-treatment		35.18	13.77		.53*
GSRS-IBS at follow-up		32.58	12.71	.53*	
*Demographic variables*					
Sex (male/female)	67/12			.14	-.17
Age		34.25	9.25	-.05	.03
Duration of IBS		13.70	11.10	.06	.07
University studies (yes/no)	52/27			-.19	-.04
*Predictor variables*					
GSRS-IBS at pre-treatment		47.74	11.00	.50*	.30*
Study group (treatment/waiting list)	39/40			-.20	-.05
Any anxiety disorder (yes/no)	39/40			.15	.16
Major depressive disorder (yes/no)	15/62			.12	.27*
MADRS-S		11.20	7.99	.20	.36*
SSP-PSTA		2.56	0.69	.07	.17
SSP-STA		2.52	0.58	.03	.03
SCL-SOM		13.52	9.19	.17	.18
VSI		42.92	17.42	.21	.28*
CSQ-CAT		14.72	7.78	.35*	.32*
ASI		19.24	12.01	.25*	.17
IBS-QOL		52.45	20.57	-.41*	-.31*
SDS		12.13	8.28	.37*	.26*

*p < .05 for bivariate correlation between predictor and predicted GSRS-IBS score. *GSRS-IBS* Gastrointestinal Symptom Rating Scale – IBS Version, *MADRS-S* Montgomery Åsberg Depression Rating Scale - Self Report, *SSP-STA* Swedish Universites Scales of Personalities - Somatic Trait Anxiety subscale, *SSP-STA* Swedish Universites Scales of Personalities – Psychic Trait Anxiety subscale, *SCL-SOM* Symptom Checklist – Somatization subscale, *VSI* Visceral Sensitivity Index, *ASI* Anxiety Sensitivity Index, *CSQ-CAT* Coping Strategies Questionnaire – Catastrophizing subscale, *IBS-QOL* Irritable Bowel Syndrome Quality of Life Instrument, *SDS* Sheehan Disability Scales.

### Measures

All self-report measurements in the study were completed online. Online assessment is considered a well-validated form of administration of self-assessments and has been shown to produce results similar to pen-and-paper administration [35].

#### Outcome

The Gastrointestinal Symptom Rating Scale –IBS version (GSRS-IBS) [36] was used as the outcome measure in this study. Because IBS symptoms vary considerably over time, the GSRS-IBS was administered weekly during three weeks at pre-treatment and post-treatment and four weeks at follow-up. The weekly average score at each assessment point was used to get reliable estimates of the participants’ symptom levels.

#### Measures of psychological distress

During the inclusion procedure in the original study all participants underwent psychiatric diagnostic assessment using the Mini-International Neuropsychiatric Interview (MINI) [37]. The MINI is a structured diagnostic interview that identifies DSM-IV Axis-I disorders. The interviews were performed by advanced graduate students in their final year of the Swedish psychology program and a clinical psychologist. All interviewers were trained in using the MINI and diagnosing psychiatric disorders.

The Montgomery Åsberg Depression Rating Scale - Self report (MADRS-S) [38] was used to measure depressive symptoms. The somatic trait anxiety (SSP-STA) and psychic trait anxiety (SSP-PSTA) are two subscales of Swedish Universities Scales of Personalities [39] and were used to measure tendencies to feeling tense, restless, and worried. To measure somatization, i.e., the tendency to experience somatic symptoms in response to psychological distress, we used the somatization subscale of the Symptom Checklist-90 (SCL-SOM) [40]. The SCL-SOM measures the severity of different somatic symptoms during the last week, e.g., headache, dizziness, pain, nausea, and weakness.

#### Measures of GSA and related constructs

The Visceral Sensitivity Index (VSI) [31], was used to measure GSA, i.e., heightened anxiety and worry about visceral sensation and IBS-associated situation (such as not being near a toilet) and behavioral attempts to avoid these stimuli. We used the catastrophizing subscale of the Coping Strategies Questionnaire, which measures the tendency to perceive pain sensation as catastrophic and unbearable (CSQ-CAT) [41]. In the present study, the participants were instructed to report their reactions also to other IBS symptoms such as constipation, diarrhea, and bloating, in addition to pain. The Anxiety Sensitivity Index (ASI) [42] was used to measure the degree to which participants believed that symptoms of anxiety and arousal could have negative consequences, such as illness, embarrassment, or increased anxiety.

#### Measures of disability

The Irritable Bowel Syndrome Quality of Life Instrument (IBS-QOL) [43] was used to assess the impact on quality of life for patients with IBS in several domains, including symptom interference with activity and impact on relationships. The Sheehan Disability Scales (SDS) [44] were used to assess symptom induced disability in three domains: social, work, and family.

### Treatment

The treatment consisted of a 10-week ICBT-protocol based on exposure and mindfulness exercises. Numerous studies have demonstrated that cognitive behavioral treatments for many somatic and psychiatric disorders can be delivered over internet with effects similar to face-to-face treatment [45]. The ICBT protocol in the present study was mainly based on the principle of behavioral exposure, i.e., participants provoked IBS symptoms and reduced their avoidance of stimuli that induced symptoms or worry about symptoms. The proposed mechanism was that the exposure exercises, aided by mindfulness exercises, would break the cycle of symptom and disability maintenance that the GSA behavioral pattern constitutes, leading to improvement in symptoms and quality of life. The original report contains a detailed description of the treatment program and results [27].

### Assessment

All self-assessments were collected before treatment start. The GSRS-IBS was also completed at post-treatment and 15–18 months follow-up. The telephone interviews, SSP-STA, and SSP-PSTA were administered at the screening phase of the study, i.e. 1 week to 3 months before treatment start for the original treatment group and about 3–6 months before treatment start for the waiting list.

### Analysis

All statistical analyses were performed using SPSS 21. The two samples, one with participants who received ICBT directly and the other comprising participants who received ICBT after 10 weeks on waiting list, were pooled into a single sample to increase statistical power. This was deemed acceptable, as there were no statistically significant differences between the groups on any of the demographics (age, sex, and duration of IBS), predictors, or dependent variables, i.e., the post-treatment and follow-up scores on the GSRS-IBS. The analyses were conducted in two steps. First, the GSRS-IBS post-treatment and follow-up scores were correlated with the demographic and predictor variables. Second, all demographic and predictor variables that were significantly correlated with the post-treatment or follow-up GSRS-IBS scores were entered into two separate multiple linear regression models, using the post-treatment and the follow-up GSRS-IBS scores as the dependent variable. The GSRS-IBS pre-treatment score was included in both regression models to adjust for initial baseline level and account for reliability of the measurement. This procedure often yields results equivalent to using residualized change scores in the prediction of treatment outcomes [46] and is superior to dichomotization (such as median split) in terms of increased power and decreased risk of type-I errors [46,47]. We also examined the impact of outliers and multicollinearity on the results. The power of the analysis to detect a predictor variable with a moderate effect sixe (f^2^ = .15 or higher) was 93% with alpha-level set at .05.

## Results

### Descriptive statistics

Table 1 displays descriptive statistics of the demographic, predictor, and outcome variables. As can be seen in Table 1, the mean GSRS-IBS score decreased from 47.74 at pre-treatment to 35.18 at post-treatment and 32.58 at follow-up for the whole sample. According to the MINI interview before randomization, 42 participants had diagnosable psychiatric condition, 15 participants were diagnosed with major depressive disorder and 39 participants with an anxiety disorder (the most common were agoraphobia, *n* = 21, generalized anxiety disorder, *n* = 18, social anxiety disorder, *n* = 10, and panic disorder, *n* = 10).

### Bivariate correlations

The bivariate correlations between the demographic/predictor variables and the outcome measures are displayed in Table 1. The pre-treatment scores on the GSRS-IBS were significantly correlated with both the post-treatment and follow-up scores on the same measure. In addition, the CSQ-CAT, ASI, IBS-QOL, and SDS were significantly correlated with the post-treatment GSRS-IBS score and the diagnosis of major depressive disorder as well as scores on the MADRS-S, VSI, CSQ-CAT, IBS-QOL, and SDS were significantly correlated with the follow-up GSRS-IBS score. With the exception of IBS-QOL, all significant correlations were positive. This means that more severe symptoms or disability were correlated with worse outcome on the GSRS-IBS. None of the demographic variables were related to outcome.

### Regression analyses

All predictors that were significantly correlated with the outcome were entered into multiple linear regression analyses, separate for the post-treatment and follow-up scores of the GSRS-IBS. The results of the regression analyses are presented in Table 2. None of the predictor variables remained significantly associated with the post-treatment or follow-up scores on the GSRS-IBS, with the exception of the pre-treatment GSRS-IBS score, which significantly predicted the post-treatment GSRS-IBS score. The models accounted for 28.7% (F(5,70) = 5.64, p< .001) and 19.5% (F(7,66) = 2.28, p = .039) of the variances in GSRS-IBS post-treatment and follow-up scores, respectively.

**Table 2:** Regression models predicting GSRS-IBS scores at post-treatment and follow-up

	**Coeff**	** CI-**	** CI+**	*** β***	*** t***	*** p***	**Partial *r***
***Prediction of GSRS-IBS at post-treatment. R = .536. R***^***2***^ ***= .287. p < .001***							
Intercept	10.66	−18.52	39.84		0.73	.469	
GSRS-IBS pre-treatment	0.51	0.19	0.83	.40	3.18	.002	.36
CSQ-CAT	−0.03	−0.58	0.51	-.02	−0.10	.921	-.01
ASI	−0.01	−0.29	0.27	-.01	−0.08	.939	-.01
IBS-QOL	−0.05	−31.64	21.48	-.08	−0.38	.704	-.05
SDS	0.27	−0.23	0.75	.16	1.08	.284	.13
*Prediction of GSRS-IBS at follow-up. R = .441. R*^*2*^ *= .195. p = .039*							
Intercept	12.75	−25.75	51.26		0.66	.511	
GSRS-IBS pre-treatment	0.21	−0.12	0.54	.18	1.25	.215	.15
Major depressive disorder	5.31	−2.32	12.94	.17	1.39	.169	.17
MADRS-S	0.32	−0.15	0.78	.20	1.37	.177	.17
VSI	0.08	−0.22	0.38	.11	0.53	.597	.07
CSQ-CAT	−0.01	−0.59	0.58	.00	−0.02	.985	.00
IBS-QOL	0.03	−30.83	36.68	.05	0.17	.863	.02
SDS	0.04	−0.46	0.54	.03	0.17	.866	.02

CI-: 95% Confidence interval, lower bound. CI + 95% Confidence Interval, upper bound. *GSRS-IBS* Gastrointestinal Symptom Rating Scale, IBS version, *MADRS-S* Montgomery Åsberg Depression Rating Scale - Self Report, *VSI* Visceral Sensitivity Index, *ASI* Anxiety Sensitivity Index, *CSQ-CAT* Coping Strategies Questionnaire – Catastrophizing subscale, *IBS-QOL* Irritable Bowel Syndrome Quality of Life Instrument, *SDS* Sheehan Disability Scales.

Several alternative models were evaluated in sensitivity analyses. (1) When testing for possible multicollinearity, the GSRS-IBS and VSI scores were found to be highly correlated, *r* = .84. Because the model predicting follow-up scores included both these measures, alternative models were tested with either the VSI or IBS-QOL removed. (2) One outlier who scored more than 3 standard deviations above the sample mean on the GSRS-IBS at both post-treatment and follow-up was identified, and all analyses were rerun without this outlier. (3) The analyses were also rerun separately for the original study’s treatment group and waiting list group. (4) Each of the predictors that showed a significant bivariate correlation with the outcome measures were investigated separately by entering them into regression models that only controlled for the pre-treatment GSRS-IBS score (i.e., no other predictors were entered into the model). None of these alternative models gave substantially different results.

## Discussion

In this study, we aimed to investigate possible predictors of symptomatic improvement in a previously conducted trial in which study participants with IBS underwent ICBT, which targeted GSA by exposure exercises and mindfulness training. The investigated predictor domains were psychological distress, GSA, and disability. The original reports showed that the ICBT was effective in reducing IBS symptoms and that improvements were maintained over a long follow-up time [27,34]. Although several of the prognostic factors were correlated with the symptomatic outcome, none remained a significant predictor when entered into a multiple regression model that included the pre-treatment symptom levels. Thus, the results in this study largely follow the same pattern of previous prediction studies in the IBS field, with no variable appearing as a clear and consistent predictor of treatment outcome. This is important information from a clinical perspective as it means that the clinician could, considering that ICBT leads to symptom reduction, offer the treatment regardless if the patient is an older woman with comorbid anxiety and severe IBS symptoms or a younger man without psychiatric comorbidity and moderate IBS symptoms.

Because the treatment under investigation in this study did not target comorbid psychological distress, we did not expect the presence of comorbid psychological distress to be predictive of treatment success. However, it is interesting to note that the proposed maintaining mechanisms of the psychiatric disorders found in this sample (primarily depression, panic disorder, social anxiety disorder, and generalized anxiety disorder) are similar to how GSA has been suggested to maintain IBS. Cognitive behavioral models emphasize how threat-orientation, worry, and avoidance related to disorder-specific stimuli serve to maintain these disorders e.g., [48]. Given the high comorbidity between common psychiatric disorders, this behavioral pattern has been suggested to be a shared causal factor between many psychiatric disorders, labeled emotional or experiential avoidance [49]. Similarly, there is a large overlap between IBS and other functional somatic disorders, e.g., fibromyalgia, chronic pain, and chronic fatigue syndrome [8], and worry, fear, hypervigilance, and avoidance in response to the disorder-specific symptoms have also been suggested to be important maintaining factors in these disorders as well as IBS [50]. Thus, emotional or experiential avoidance seems to be a behavioral phenomenon that unifies these functional somatic and psychiatric disorders and GSA may simply be a manifestation of this behavioral pattern in the context of IBS. Therefore, a patient who presents with both depression and IBS could be said not to be suffering from two comorbid disorders, of which one is especially suitable for psychological treatment, but rather to be displaying an avoidant behavioral pattern in response both to bowel symptoms and the routine demands of life, while a patient with IBS but without psychiatric comorbidity only displays this behavioral pattern in response to bowel symptoms. In essence, this putative explanation of the psychiatric comorbidity between the IBS and psychiatric disorders does not stipulate that psychiatric disorders should be present or absent for a GSA-focused treatment to work for IBS.

We have recently demonstrated that change on the VSI is a mediator of treatment effect on IBS symptoms in the exposure-based treatment for IBS [6]. This finding is in line with the proposed role of GSA as a maintaining factor in IBS and it could therefore be expected that patients who score high on the VSI would benefit more from the treatment than patients with low VSI scores. However, we did not find that pre-treatment scores on the VSI predicted symptomatic improvement. The stable placebo response in IBS may be a possible explanation for this finding. Reviews have shown that about 40% of patients with IBS respond to placebo treatments [51]. It could be hypothesized that even if participants with high levels of GSA, as measured by the VSI, responded favorably to the exposure exercises, participants with lower levels of GSA could also show treatment response, but this response would be due to unspecific factors rather than the specific interventions in the treatment. Even if the placebo-induced treatment response among low-GSA participants was smaller than the intervention-specific response for high-GSA participants, these two “competing” mechanisms of treatment response would mean reduced power to detect the influence of pre-treatment GSA levels on symptomatic reduction.

This reasoning is partly supported by our recent study of mechanisms of change. There we observed that in the exposure treatment, engagement in exposure and mindfulness exercises was associated with improvement on the VSI (which in turn was associated with improvement on the GSRS-IBS) but in the comparison arm, a stress management condition, engagement in strategies such as relaxation and lifestyle changes was not associated with change in any measured mechanism [6]. Thus, the effect in the stress management arm may have been due to non-specific factors rather than specific symptom maintaining factors in IBS. Also, it is important to underscore that it is not necessary for GSA to predict outcome even if it mediates the treatment effect. It could be that reducing GSA leads to decreased symptoms, but that participants make similar GSA reductions, regardless of initial baseline levels of GSA. If this was the case, than there would be no strong predictive effect of GSA on outcome as assessed in the present study.

There were some important limitations that need to be considered when interpreting the results from this study. First, this was a secondary analysis on data from a study that was not designed to address the questions posed in this article. The lack of predictive value of the measures may be due to the nature of the sample where all participants were self-referred and therefore prepared to engage in a psychological treatment. A larger and more representative sample could have presented with a larger variation in pre-treatment characteristics and outcome and thus revealed true differences between low and high scoring groups that were not detectable in the sample under investigation. Second, a larger sample could also have provided more power to detect meaningful effects of the pre-treatment characteristics on the GSRS-IBS at post-treatment and follow-up. For example, the difference between depressed and non-depressed participants on the GSRS-IBS follow-up score was 5.31 with a 95% confidence interval of −2.32 to 12.94 (*p* = .169), controlling for all other predictors. As the follow-up score on the GSRS-IBS was 32.58, the non-significant difference of 5.31 points between depressed and non-depressed could actually represent a meaningful difference in outcome (especially when considering that by convention, the GSRS-IBS is scored between 13 and 91, rather than between 0 and 78, which in essence means that all difference scores should be compared with absolute scores subtracted with 13, i.e., a difference of 5.31 compared with a zero-based mean follow-up score of 19.58). Third, most of the psychiatric assessments were made by final year psychology students who assessed the presence of psychiatric diagnosis in interviews conducted over the telephone. The proportion of participants with a psychiatric disorder in this study, 42 of 79 (53%), is close to the common approximation that about half of the IBS population has a diagnosable psychiatric disorder [52]. Still, with inexperienced assessors, individual participants may have been misclassified, limiting the validity of the prediction analyses based on the presence of psychiatric diagnosis. Fourth, the VSI may not be a measure that adequately captures the GSA phenomenon as it pertains to the exposure-based treatment. Of the 13 items on the VSI, only two measure behavioral responses to the IBS in terms of avoidance and safety behaviors while the remaining 11 items measure symptom-related worry, preoccupation, and sensitivity [31]. Because the exposure-based treatment mainly targets excessive avoidance and control behaviors, the VSI may not measure the behavioral aspects of GSA that are most relevant for prediction of outcome of this treatment. The Irritable Bowel Syndrome-Behavioral Responses Questionnaire (IBS-BRQ) [53], which only measures IBS-related avoidance and control behaviors, could have proved to be a better measure of suitability for the treatment. In fact, high scores on the IBS-BRQ have been shown to predict better response to CBT for IBS [13]. However, that specific CBT protocol was not exposure-based [54] and the predicted outcomes were work and social adjustment and not symptomatic improvement. Therefore, the results from the prediction study that included the IBS-BRQ may not be comparable with this study.

Given the results of this and previous prediction studies, it is possible that the search for individual characteristics as stable predictors of treatment outcome is futile. It is also important to note that even if variation in specific individual characteristics were found to be associated with treatment outcome, most likely they will be associated with an increased likelihood that symptomatic improvement will occur rather than be binary determinants of whether an individual participant will respond or not. An important venue of future research could then be to focus on other potential predictors of outcome related to the therapeutic process rather than pre-treatment characteristics, for example treatment expectancy, treatment adherence, and treatment response patterns. The outcome of ICBT for fibromyalgia, social anxiety disorder, severe health anxiety disorder has been shown to be associated with the participants’ adherence to treatment [55-57] and early response during the course of face-to-face CBT for IBS has been associated with better long-term improvement [58].

## Conclusions

The findings of the present study suggest that ICBT for IBS has similar effects on symptoms regardless of demographic characteristics and psychiatric comorbidity in a self-referred sample. Considering the large prevalence of IBS and the many different psychological treatment protocols that have been developed, the need for more knowledge about how to match patients to treatment remains.

## Abbreviations

ASI: Anxiety sensitivity index; CBT: Cognitive behavior therapy; CSQ-CAT: Coping strategies questionnaire – catastrophizing subscale; GSA: Gastrointestinal symptom-specific anxiety; GSRS-IBS: Gastrointestinal symptom rating scale – IBS version; IBS-QOL: Irritable bowel syndrome quality of life instrument; IBS: Irritable bowel syndrome; ICBT: Internet-based cognitive behavior therapy; MADRS-S: The montgomery asberg depression rating scale - self report; SCL-SOM: Symptom checklist – somatization subscale; SDS: Sheehan disability scales; SSP-STA: Swedish Universites scales of personalities - somatic trait anxiety subscale; SSP-STA: Swedish Universites scales of personalities – psychic trait anxiety subscale; VSI: Visceral sensitivity index.

## Competing interests

The authors declare that they have no competing interests.

## Authors’ contributions

BL conceived the idea of the study, wrote the treatment manual, supervised the treatment, conducted the analyses, and drafted the manuscript. ErA interviewed the participants and drafted the manuscript. PL supervised the recruitment process. JML provided expertise in interpretation of the results. KG, KM, JN, and KR collected outcome data and performed preliminary data analysis. KG and JN also treated participants and KM and KR also interviewed participants. EvA assisted in interpretation of the results and drafted the manuscript. TH supervised the preliminary data analysis. HH supervised the data analysis and drafted the manuscript. EH participated in the treatment design, co-authored the treatment manual and drafted the manuscript. All authors contributed to the manuscript and interpretation of the results and read and approved the final manuscript.

## Pre-publication history

The pre-publication history for this paper can be accessed here:

http://www.biomedcentral.com/1471-230X/13/160/prepub
